# Clinical Outcomes of Cardiac Rehabilitation in Women with Coronary Artery Disease—Differences in Comparison with Men

**DOI:** 10.3390/jpm12040600

**Published:** 2022-04-08

**Authors:** Katarzyna Szmigielska, Anna Jegier

**Affiliations:** Department of Sports Medicine, Medical University of Lodz, Pomorska 251, 92-213 Łódź, Poland; anna.jegier@umed.lodz.pl

**Keywords:** women, cardiac rehabilitation, physical capacity

## Abstract

This study evaluated the clinical outcomes of cardiac rehabilitation (CR) in women with coronary artery disease (CAD) in comparison to men. Methods: Patients after acute coronary syndrome or after revascularization procedures (106 women, 180 men) were consecutively admitted to a comprehensive outpatient CR program, comprising of 45-min ergometer interval training three times a week for eight weeks. The training intensity was determined on the basis of training heart rate, calculated following an exercise test. Patients were divided into subgroups according to age (≤55, >55 years), BMI (<25, ≥25 kg/m^2^), left ventricular ejection fraction (LVEF; ≤40%, 41–49%, ≥50%), and number of affected coronary vessels. Results: After eight weeks, exercise capacity increased significantly by 0.6 ± 0.77 MET (women) and by 1.0 ± 0.74 MET (men). The greatest benefit was observed in men, women under 55 years, women with LVEF 41–49%, and women with single-vessel CAD. An outpatient CR program appears less beneficial for women, especially those over 55 years, with two or three coronary vessels affected with atherosclerosis or with LVEF > 50%. In women with CAD, eight weeks of 45-min interval training, with sessions three times a week, is insufficient to improve exercise capacity to an extent that is considered a predictor of mortality risk reduction.

## 1. Introduction

Ischaemic heart disease (IHD) is the most common cause of death in the world [1]. The development of invasive procedures in the treatment of coronary artery disease (CAD) has greatly reduced the mortality after an acute coronary syndrome (ACS). Cardiac rehabilitation (CR) after ACS is essential to further reduce this risk. Cardiac rehabilitation based on regular physical activity leads to many benefits, including improvement of VO_2MAX_, exercise capacity and endothelial function, lowering of blood pressure and body weight, and modification of the blood lipid profile. It is also associated with an improvement in quality of life and a reduction in mortality risk by up to 26% [2,3,4,5,6,7]. It has been estimated that an increase in exercise capacity by 1 metabolic equivalent (MET) results in a fall in all-cause mortality from 8% to even 26% [5,6]. The morbidity and mortality of CAD are different in men and women. The average woman with ACS is usually older, has more comorbidities, and receives suboptimal treatment compared to her male counterpart [8,9,10,11]. Women may also have more diffuse atherosclerosis in their coronary arteries. Ischemic heart disease in women is not only atherosclerotic obstructive CAD, but can also be associated with coronary microcirculation dysfunction, vasomotor abnormalities, endothelial dysfunction, stress-induced cardiomyopathy, or spontaneous dissection of the coronary artery [12,13]. The aim of this observational study was to evaluate the clinical outcomes of cardiac rehabilitation in women with CAD in comparison to men, with regard to their age, Body Mass Index (BMI), left ventricular ejection fraction (LVEF), and number of vessels affected by atherosclerosis, as well as to determine which group of patients had a greater benefit from cardiac rehabilitation.

## 2. Materials and Methods

### 2.1. Participants

The study covered 286 patients, 106 female and 180 male, aged 31 to 85 years (mean age 65.05 ± 7.2 and 60.5 ± 10.5 years, respectively) after ACS, coronary artery bypass surgery (CABG), or percutaneous coronary interventions (PCI), who were prospectively and consecutively admitted to an outpatient comprehensive cardiac rehabilitation program for eight weeks. The mean time following PCI was 22.3 ± 9.1 days and CABG was 26.4 ± 8.5 days. The study was conducted in the Outpatient Rehabilitation Center of the Clinical University Hospital.

All the patients enrolled to the study were treated with β-blockers, angiotensin converting enzyme inhibitors, or angiotensin receptor blockers, statins, and antiplatelet agents. All the examined patients had an optimal and stable pharmacological treatment. Any changes in pharmacotherapy during the rehabilitation program resulted in exclusion from the study. The statistical analysis included only patients who completed the entire eight-week program of CR.

### 2.2. Program of Cardiac Rehabilitation

Three times a week for 45 min, the patients participated in interval trainings on cycle ergometers (Ergoselect II 100/200 Ergoline Reha System GmbH, Schiller, Switzerland). Patients’ electrocardiographic values and blood pressure were continuously monitored during the training sessions. In the first part of the training session, the workloads were increasing, while in the second part they were decreasing, alternating a four-minute workload with two minutes of active restitution. The peak exercise intensity was determined on the basis of training heart rates (THR) and assessed according to the heart rate reserve (HRR), which was individually calculated as the difference between the resting HR and the highest heart rate achieved during the exercise test [14]. The formula to calculate the THR was

THR = resting HR + (0.5 to 0.7) HRR(1)

The training intensity was adjusted so that the patient’s heart rate achieved, but not exceeded, the calculated training heart rate and it ranged from 12 to 14 points on the Borg scale [15]. In addition to 24 exercise training sessions, the comprehensive cardiac rehabilitation program consisted of a health education on cardiovascular risk factors, lifestyle modification including physical activity, healthy diet, and psychological support in coping with nicotine addiction and stress management.

### 2.3. Data Collection

At the beginning and after eight weeks of cardiac rehabilitation, all the participants underwent a physical examination, an exercise test, and fasting blood tests.

Body weight rounded to the nearest 50 g and body height rounded to the nearest millimeter were determined with standard anthropometric methods: using a digital, medical scale (Radwag WPT 100/200) and a stadiometer (GMP, Switzerland), respectively. Waist circumference, with an accuracy of 10 mm, was measured at the midpoint between the iliac crest and the lowest rib. Body mass index (BMI) was calculated as weight/height^2^ (kg/m^2^). All the participants underwent an electrocardiogram at rest in the supine position and a physical examination including blood pressure (BP) measurements.

**Scheme 1:**
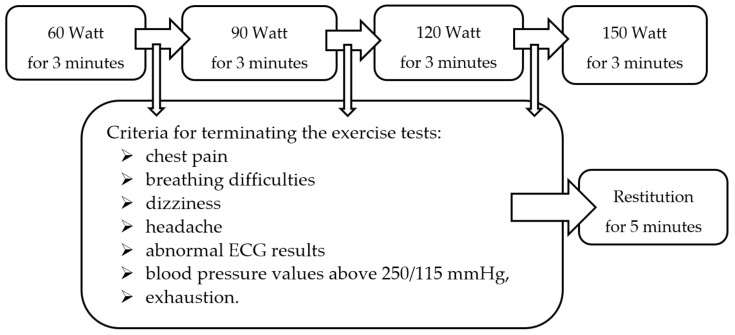
Scheme of the multistage, symptom-limited exercise test on the cycle ergometer.

Each subject underwent a multistage, symptom-limited exercise test on the cycle ergometer Ergoselect II 100/200 with continuous 12-lead electrocardiographic monitoring using a Cardiovit CS-200 Ergo Spiro; Schiller, Switzerland. The exercise test began at 60 Watt, and the workload was gradually increased by 30 Watt every three minutes until exhaustion. When the exercise phase was completed, the participants were monitored for five minutes or longer, if necessary. The criteria for terminating the exercise tests included: clinical symptoms such as chest pain, breathing difficulties, dizziness or headache, abnormal ECG results, blood pressure values above 250/115 mmHg, or exhaustion (Scheme 1).

The rate pressure product (RPP) was calculated as heart rate (HR) x systolic BP. The resting RPP was calculated from resting values, while the RPP peak was based on the highest values obtained during the exercise test. The peak workload (Wpeak), expressed in Watt, was defined as the highest workload during exercise test. The patients’ exercise capacity was also expressed as the highest value in METs reached during exercise test. Also, at the last stage of the exercise test, the rating of perceived exertion was assessed according to the 20-point Borg scale [15].

The serum glucose concentration, total cholesterol, triglycerides and high-density lipoprotein cholesterol (HDLc), and low-density lipoprotein cholesterol (LDLc) were assessed using the enzymatic calorimetric method with Cormay reagents.

### 2.4. Echocardiography

After the 8 weeks of the rehabilitation program, the M mode and two-dimensional echocardiography with Doppler technique using VIVID7 (General Electric Healthcare, Boston, MA, USA) was performed by the same researcher. Data were obtained at rest using a 3.5-MHz linear transducer. The measurements were taken and analyzed according to the guidelines of the American Society of Echocardiography [16]. In order to assess the left ventricular function, the LVEF, the presence of segmental systolic dysfunction, and left ventricular hypertrophy and its enlargement were measured and assessed.

The data from echocardiography performed after eight weeks of cardiac rehabilitation were compared to the results of echocardiographic examination performed during the hospitalization that was connected with ACS, PCI, or CABG (which was an indication for CR). To assess whether all patients benefited from cardiac rehabilitation equally, they were divided into subgroups according to age (≤55, >55 years), BMI (<25, ≥25 kg/m^2^), LVEF (≤40%, 41–49%, ≥50%) [4], and the number of coronary vessels affected with atherosclerosis (single-vessel, two-vessel, and three-vessel disease).

### 2.5. Statistical Analysis

In the analysis of the differences between the groups, the U Mann–Whitney test was used for continuous variables without a normal distribution and a two-sample Student’s *t*-test was used to compare the means of continuous variables with a normal distribution. To compare initial and final values in each group, a Wilcoxon test and paired *t*-test were applied for nonparametric and parametric data, respectively. The measured values were presented as the median with interquartile range and as mean ± standard deviation, if appropriate. The difference was considered statistically significant when the *p* value was lower than 0.05. The statistical analysis was performed with Statistica software, Version 13.1, USA.

## 3. Results

Women in the analyzed group were significantly older than examined men (65.05 ± 7.2 years and 60.05 ± 10.5 years, respectively). The study participants were not significantly different in terms of clinical history, cardiovascular risk factors, or frequency of coexisting diseases. Women more commonly had a single-vessel form of CAD, whereas a three-vessel CAD was more commonly found among men; these differences were statistically significant (Table 1).

Women admitted for the rehabilitation program had a higher value of LVEF, less frequent segmental systolic left ventricular dysfunction, left ventricular hypertrophy, and left ventricular enlargement in comparison to the group of men at the beginning of CR (Table 2).

The glucose serum concentrations and triglycerides were similar in groups of women and men, while the total cholesterol, LDL cholesterol, and HDL cholesterol were statistically significantly higher in women than in men at the beginning of CR program. Apart from the increase in triglycerides in the male group, none of the parameters changed within eight weeks of CR (Table 3).

Post-CR BMI was significantly higher in male patients when compared to their pre-CR BMI, while BMI in the female group remained unchanged. Waist circumference did not change in both examined groups after eight weeks of CR (Table 4).

Among hemodynamic parameters at rest, only resting heart rate in the female group did not significantly change after 8 weeks of CR. Resting heart rate among male patients and resting systolic and diastolic arterial blood pressure were significantly decreased in both groups after eight weeks of CR compared to the baseline measurements.

Exercise capacity expressed in MET also improved significantly after 8 weeks of CR in both examined groups. The increase in exercise capacity by 0.6 ± 0.77 MET, (the median 0.0 MET) was observed in the female group, while in the male group, the mean increase in exercise capacity was 1.0 ± 0.74 MET, (the median 1.1 Met) (Table 4).

LVEF significantly improved and segmental systolic left ventricular dysfunction decreased after 8 weeks of CR in both examined groups. The other echocardiographic parameters remained unchanged (Table 2).

The results of the statistical analysis of changes in exercise capacity in the created subgroups according to age, BMI, LVEF, and the number of coronary vessels affected with atherosclerosis are presented in Table 5.

### 3.1. Age Subgroups

**Figure 1:**
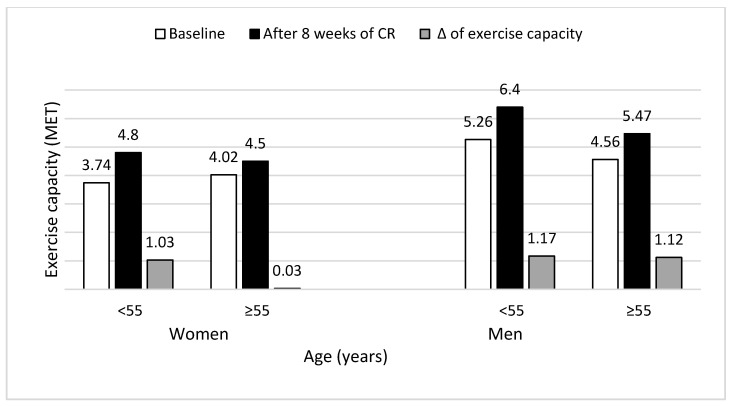
The exercise capacity of examined women and men with CAD at baseline and after 8 weeks of CR program in age subgroups.

Only nine women under the age of 55 years participated in the study. There were significantly more men than women in this age group. The increase of exercise capacity was more pronounced in women under 55 years of age (by 1.03 MET) in comparison to women older than 55 years (by 0.03 MET). In the group of men, regardless of age, the improvement in exercise capacity was similar (by 1.17 and 1.12 MET respectively) (Table 5, Figure 1).

### 3.2. BMI Subgroups

**Figure 2:**
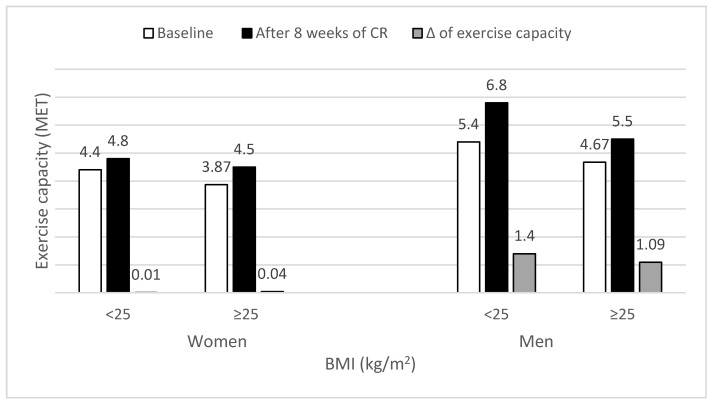
The exercise capacity of examined women and men with CAD at baseline and after 8 weeks of CR program in created BMI subgroups.

The improvement in exercise capacity in both subgroups of women, with BMI <25 kg/m^2^ and ≥25 kg/m^2^, was slight, but still significant, (by 0.01 MET and by 0.04 MET, respectively). Greater improvement was shown in both men subgroups compared to women (by 1.4 MET and by 1.09 MET, respectively) (Table 5, Figure 2).

### 3.3. LVEF Subgroups

**Figure 3:**
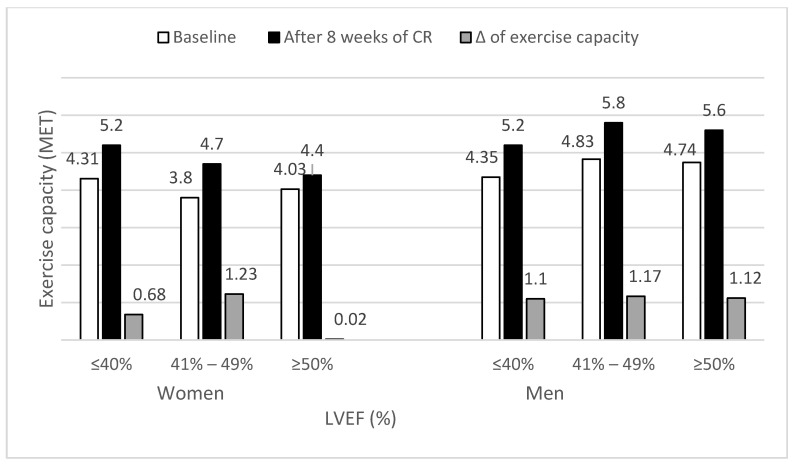
The exercise capacity of examined women and men with CAD at baseline and after 8 weeks of CR program in created subgroups related to LVEF.

There were only seven women with reduced left ventricular ejection fraction (LVEF ≤ 40%), and the exercise capacity did not change after CR in this group. A significant increase of exercise capacity by 1.23 MET was recorded only in women with LVEF 41–49%, while in woman with LVEF≥ 50%, exercise capacity increased by 0.02 MET. In all three male subgroups, exercise capacity increased similarly from 1.1–1.17 MET (Table 5, Figure 3).

### 3.4. Subgroups Created Due to the Number of Coronary Vessels Affected with Atherosclerosis

**Figure 4:**
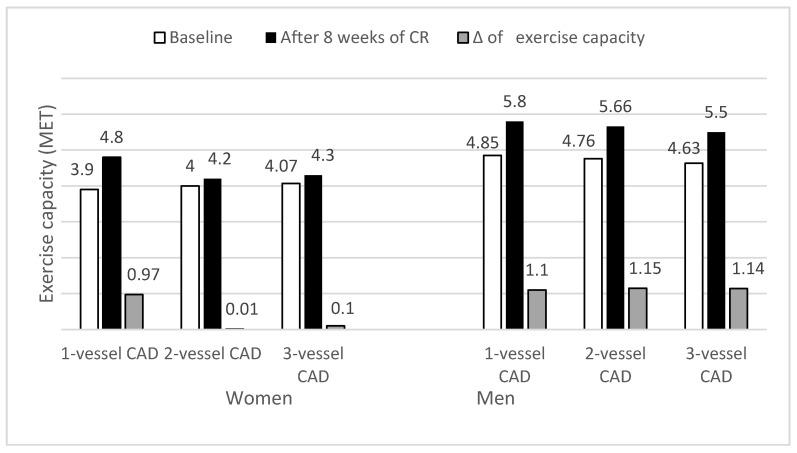
The exercise capacity of examined women and men with CAD at baseline and after 8 weeks of CR program in subgroups created due to the number of coronary vessels affected with atherosclerosis.

In the female subgroups the exercise capacity was found to have risen by 0.97 MET only in group with single-vessel CAD. A slight but significant increase was observed in women with two-vessel CAD and no statistical change in exercise capacity was seen in women with three-vessel disease. In the male subgroups, a significant increase in exercise capacity from 1.1–1.15 MET was identified, regardless of the number of coronary vessels involved (Table 5, Figure 4).

## 4. Discussion

According to guidelines, regardless of gender, CR is a class I level A recommendation for patients after myocardial infarction, CABG, or PCI, and rehabilitation should be based on aerobic exercise training of moderate to high intensity for a minimum of 30 min at least 3 times a week [3]. Numerous studies have shown an improvement in the survival of patients who underwent cardiac rehabilitation after ACS [2,5,6,7]. The most important finding of our investigation is that an increase in exercise capacity after eight weeks of CR in the female group was lower than in the male group (by 0.6 ± 0.77 MET and 1.0 ± 0.74 MET, respectively).

Many studies have shown that exercise capacity is a stronger predictor of mortality risk than traditional risk factors such as smoking, hypertension, hyperlipidemia, obesity, and type 2 diabetes. Moreover, exercise capacity is considered to be a more powerful predictor of risk than other exercise test variables, such as ST segment depression, exercise-related symptoms, and hemodynamic responses [17,18]. According to previous research, the increase of exercise capacity by each 1 MET was connected with a reduced risk of cardiovascular mortality by 12% [5], by 16.1% [19], and even by 26% [6]. In our investigation, although the exercise capacity of examined women improved, the increase was lower than 1 MET and was statistically lower when compared to men. Previously published studies showed a significant increase in exercise capacity after CR regardless of gender [20,21,22,23,24,25,26], although there are not many studies available comparing the effect of CR on exercise capacity in women and men, and the outcomes of these studies are inconsistent. Some studies reported that the improvements in exercise capacity after CR are greater in men than women. [25,26] Other studies showed no difference in functional capacity improvements between men and women after CR [27,28]. In these two studies, which showed a similar increase in exercise capacity in men and women, exercise capacity was assessed on the basis of 6-Minute Walk Test (6MWT), not an exercise test on a treadmill or cycle ergometer with estimation of METs or directly measured VO2max. This might be a possible explanation of the inconsistent results of studies designed differently.

In our study, significantly more men than women participated in the CR program. Many studies showed that women are less likely to be referred and to participate in CR for various reasons, leading to higher mortality rates among women who are not referred to CR [3,8,9,10,11,29].

Moreover, our study involved women who were significantly older than men (*p* = 0.00052). There were fewer women than men in the under 55 age group. This may be due to the fact that in the premenopausal period, secreted estrogens reduce the risk of coronary heart disease [30,31]. However, such conclusions have not been confirmed by randomized clinical trials for prevention of atherosclerotic cardiovascular disease [32,33]. In our investigation, the examined women, although older, had less damage to the coronary vessels and the heart muscle than examined men. Women had single-vessel CAD more frequently, and three-vessel CAD was more common in men. At the beginning of CR, the female group also had a higher value of LVEF, and less frequent segmental systolic left ventricular dysfunction, left ventricular hypertrophy, and left ventricular enlargement in comparison to the male group. Moreover, the total cholesterol and LDL cholesterol were significantly higher in women than in men at the beginning of CR program.

In previously published studies, women were more likely to be obese, have a worse risk-factor profile, and have depression and anxiety more often [10,11,34]. According to other published studies, women also received preventive therapy less often than men with a similar risk of atherosclerotic CAD [35]. Pharmacotherapy of hypertension or hyperlipidemia in women were often less aggressive and optimal results were less often achieved [8,9,10,11,36,37].

In our investigation, most outcomes of an eight-week CR were similar in men and women. Hemodynamic parameters measured at rest improved significantly in both groups of patients. BMI and waist circumference did not improve after eight weeks of CR in both men and women. BMI in the group of men increased slightly, but significantly. According to recommendations, to achieve bodyweight reduction, aerobic exercise training should be performed more than 150 min per week [3]. In addition, it is possible that eight weeks of 45-min training three times a week is too short a period of time to induce effective weight loss. Differences between women and men were observed in the strongest predictor indicated in previous publications: exercise capacity. Statistical analysis in the created subgroups showed that in the group of men, CR led to an improvement in exercise capacity by more than 1 MET, regardless of age, the BMI, LVEF, and the number of coronary vessels affected.

In the women who were under 55 years of age, a more pronounced increase in exercise capacity was observed (despite the small size of the group which made statistical analysis difficult). BMI had no influence on the changes of exercise capacity in women. Previously published studies also showed no impact of BMI on the increase in exercise capacity after CR, regardless of gender [38].

An increase in exercise capacity by more than 1 MET was observed only in women with LVEF 41–49% (median of increase was 1.23 MET) and by 0.97 MET in women with single-vessel CAD. In women with LVEF≥ 50%, and in women with two-vessels CAD, the increase of exercise capacity was slight (median 0.02 MET and 0.01 MET, respectively); in women with three-vessels CAD, the changes in exercise capacity were not significant. In previously published studies, left ventricular dysfunction has been found to be associated with an increased exercise capacity connected with a CR program [39,40,41]. The increase in exercise capacity appeared to be the most substantial in the patients with the lowest exercise capacity (measured by oxygen intake pVO2 < 20 mL/kg/min) [41]. Furthermore, clinical trials and meta-analyses have shown an improvement of exercise capacity in people with heart failure with a reduced ejection fraction after exercise-based rehabilitation [42,43]. In the analysis of the created subgroups, the greatest benefit from CR was observed in men in all subgroups, women under 55 years, women with LVEF 41–49%, and women with single-vessel CAD.

CR appears less beneficial for women over 55 years of age, with LVEF >50%, and with two or three coronary vessels affected with atherosclerosis.

Eight weeks of aerobic training in a CR program might not have been sufficiently long to induce adequate changes in exercise capacity in the group of women with CAD because the origin of ischemia in the heart muscle is different in women than in men [10,11]. A longer duration of a CR program might be more effective in women. In the study analysing three months of aerobic exercise training, a similar exercise training response in women and men with CAD has been shown [44]. The duration is not the only parameter of aerobic training that might influence CR outcomes. The training volume might also have an impact on the outcomes of physical activity. In recently published study, the workload and duration of training sessions were significant predictors of functional capacity improvement of CAD patients who participated in CR based on treadmill exercise sessions [45]. According to the recommendations, the frequency of aerobic training should be at least three days a week, preferably six or seven days a week [3].

Programs of cardiac rehabilitation vary around the world. Some cardiac rehabilitation programs have been shown to be ineffective in improving cardiovascular risk factors, morbidity, and mortality in patients with cardiovascular diseases. It seems that in order to obtain an improvement, women, especially those over 55 years of age, with LVEF ≥50% or two or three coronary vessels affected with atherosclerosis, should undergo cardiac rehabilitation based on physical training for a longer period of time, and exercise should be performed in a larger volume. Eight weeks of the 45-min training program completed three times a week improved blood pressure at rest, LVEF, and exercise capacity; however, it was not enough to improve exercise capacity by 1 MET or more in women with CAD. A larger volume of training might be more effective in achieving better outcomes of cardiac rehabilitation. The time spend on training should be at least 150 min per week, optimally about 300 min per week [3].

### Strengths and Limitations of the Study

The novel aspect of the present study is the comparison of the impact that physical training has on exercise capacity in women in comparison to men with regard to age, BMI, LVEF, and according to the number of coronary vessels affected with atherosclerosis. The limitation of our study is the lack of a matched control group. Unfortunately, it was not possible to create the control group because it would not be ethical to refuse CR to any patient with indications, and those who refuse to participate in the rehabilitation program are reluctant to attend control visits. We realize that our study is purely observational.

## 5. Conclusions

An outpatient CR program based on regular aerobic interval training appears less beneficial for women, especially those over 55 years of age, with two or three coronary vessels affected with atherosclerosis, or with LVEF over 50%. In women with CAD, eight weeks of 45-min interval training, with sessions three times a week, is insufficient to improve exercise capacity to an extent that is considered a predictor of mortality risk reduction.

## Figures and Tables

**Table 1:** Baseline anthropometric parameters, risk factors and clinical history of studied women and men with CAD before CR.

Study Participants (*n* = 286)	Women (*n* = 106)	Men (*n* = 180)	*p*
Age (years)	65.05 ± 7.2 66 (47–83)	60.5 ± 10.5 62 (31–85)	0.00052
BMI (kg/m^2^)	28 (20.8–41.7)	28.4 (19.1–40.8)	0.52
Waist circumference (cm)	96 (70–122)	102 (81–136)	0.00001
Clinical history			
ST Elevation Myocardial Infarction STEMI (fraction)	37 (0.35)	70 (0.38)	0.54
No ST Elevation Myocardial Infarction NSTEMI (fraction)	48 (0.45)	82 (0.45)	0.96
PCI (fraction)	91 (0.85)	151 (0.84)	0.81
CABG (fraction)	10 (0.1)	30 (0.16)	0.08
Duration of CAD (years)	0 (0–20)	0 (0–27)	0.64
Single-vessel disease (fraction)	52 (0.49)	64 (0.35)	0.025
Two-vessel disease (fraction)	31 (0.29)	52 (0.28)	0.94
Three-vessel disease (fraction)	20 (0.18)	59 (0.33)	0.01
Diabetes Mellitus (fraction)	24 (0.22)	53 (0.29)	0.21
Arterial hypertension (fraction)	86 (0.81)	149 (0.83)	0.73
Smokers/exsmokers (fraction)	0/46 (0.43)	0/92 (0.5)	0.2

CAD—coronary artery disease; CR—cardiac rehabilitation; BMI—body mass index; PCI—percutaneous coronary intervention; CABG—coronary artery bypass grafting; values are presented as median and (interquartile range) or mean ± standard deviation.

**Table 2:** Echocardiographic parameters of women and men with CAD at baseline and after 8 weeks of CR program.

Study Population (286)	Women with CAD (*n* = 106)			Men with CAD (*n* = 180)		
	Baseline	After 8 Weeks of CR	*p*	Baseline	After 8 Weeks of CR	*p*
LVEF (%)	55 (35–80)	57,5 (28–80)	0.00001	52 * (20–72)	55 ** (32–85)	0.00001
Presence of left ventricular segmental systolic dysfunction (fraction)	45 (0.42)	13 (0.12)	0.00001	99 * (0.55)	42 ** (0.23)	0.00001
Presence of left ventricular hyperthrophy (fraction)	48 (0.45)	48 (0.45)	0.18	129 * (0.72)	131 ** (0.73)	0.65
Presence of left ventricular enlargement (fraction)	15 (0.14)	15 (0.14)	0.83	63 * (0.35)	63 ** (0.35)	0.29

CAD—coronary artery disease; CR—cardiac rehabilitation; LVEF—left ventricular ejection fraction; * *p* < 0.05 group of women versus group of men at baseline; ** *p* < 0.05 group of women versus group of men after 8 weeks of CR; values are presented as median and (interquartile range).

**Table 3:** The cholesterol and the glucose serum concentration in the examined women and men with CAD at baseline and after 8 weeks of CR program.

Study Population (286)	Women with CAD (*n* = 106)			Men with CAD (*n* = 180)		
	Baseline	After 8 Weeks of CR	*p*	Baseline	After 8 Weeks of CR	*p*
Total cholesterol (mmol/L)	4.1 (2.62–7.25)	4.12 (2.21–7.29)	0.1	3.55 * (2.08–8.44)	3.54 ** (2.07–9.21)	0.54
HDLc (mmol/L)	1.41 (0.69–2.29)	1.41 (0.8–2.28)	0.41	1.1 * (0.56–1.76)	1.15 ** (0.62–1.95)	0.13
LDLc (mmol/L)	2 (0.81–4.68)	1.99 (0.81–4.98)	0.29	1.71 * (0.56–6.32)	1.72 ** (0.42–6.82)	0.24
Triglycerides (mmol/L)	1.56 (0.23–4.6)	1.48 (0.63–4.75)	0.62	1.23 (0.5–4.68)	1.32 (0.5–6.82)	0.001
Glucose (mmol/L)	5.5 (4.15–15.19)	5.37 (4.1—15.1)	0.1	5.68 (2.32–13.96)	5.68 (3.32–13.96)	0.02

CAD—coronary artery disease; CR—cardiac rehabilitation; HDLc—high-density lipoprotein cholesterol; LDLc—low-density lipoprotein cholesterol; * *p* < 0.05 group of women versus group of men at baseline; ** *p* < 0.05 group of women versus group of men after 8 weeks of CR; values are presented as median and (interquartile range).

**Table 4:** Anthropometric and hemodynamic parameters and selected indices of exercise tolerance in the examined women and men with CAD at baseline and after 8 weeks of CR program.

Study Population (286)	Women with CAD (*n* = 106)			Men with CAD (*n* = 180)		
	Baseline	After 8 Weeks of CR	*p*	Baseline	After 8 Weeks of CR	*p*
BMI (kg/m^2^)	28 (20.8–41.2)	28.05 (20.8–41.7)	0.053	28.4 (19.1–40.8)	28.5 (19.4–40.3)	0.005
Waist circumference (cm)	96 (70–122)	96 (68–120)	0.29	102 (81–136)	102.5 (80–136)	0.85
HR at rest (beats × min^−1^)	70 (55–92)	71 (49–100)	0.09	73 (52–103)	71 (49–100)	0.000002
SBP at rest (mmHg)	130 (100–150)	120 (90–150)	0.000003	120 (90–150)	120 (90–160)	0.005
DBP at rest (mmHg)	80 (60–100)	75 (50–90)	0.00003	80 (60–100)	75 (50–90)	0.004
Resting RPP × 10^−2^ (beats × min^−1^ × mmHg)	88.4 (61–135)	84.5 (56–139.5)	0.00002	88.5 * (57–138.6)	82.5 (56.7–134.4)	0.000001
Peak workload (W)	60 (30–90)	90 (30–120)	0.0000001	90 (30–180)	120 (60–240)	0.000001
Peak workload (W/kg)	0.88 (0.34–1.63)	1.03 (0.38–1.73)	0.0000001	1.08 * (0.49–2.14)	1.36 ** (0.64–2.85)	0.000001
Exercise capacity (MET)	4.02 (2.19–6.6)	4.5 (2.33–6.9)	0.0000001	4.7 (2.68–8.34)	5.67 (3.2–10.8)	0.000001
Δ of exercise capacity (MET)	0.6 ± 0.77 0.0 (−1.24–3.14)			1 ± 0.74 ** 1.1 (−1, 29–3.1)		0.0019
RPPpeak × 10^−2^ (beats × min^−1^ × mmHg)	176.2 (110.4–281.6)	185.6 (112.2–297)	0.56	174.2 (66–302.5)	185.6 (110.6–295)	0.000062
RPE (points)	14 (13–17)	15 (13–17)	0.12	15 (13–18)	16 (14–17)	0.27

CAD—coronary artery disease; CR—cardiac rehabilitation; BMI—body mass index; HR—heart rate at rest (beats/min); SBP—systolic arterial blood pressure at rest (mmHg); DBP—diastolic arterial blood pressure at rest (mmHg); RPP—rate pressure product (HR × SBP); Peak workload—workload during the last stage of exercise test; RPP*peak*—rate pressure product (HR*peak* × SBP*peak*—the highest values achieved during the last stage of exercise test); RPE—rating of perceived exertion on 20-point Borg scale during the last stage of exercise test; * *p* < 0.05 women group versus men group at baseline. ** *p* < 0.05 women group versus men group after 8 weeks of CR; values are presented as median and (interquartile range).

**Table 5:** The exercise capacity of examined women and men with CAD at baseline and after 8 weeks of CR program in created subgroups.

		Women (*n* = 106)					Men (*n* = 180)				
Subgroups		*n*	Exercise capacity (MET)				*n*	Exercise Capacity (MET)			
			Baseline	After 8 Weeks of CR	Δ of Exercise Capacity	*p*		Baseline	After 8 Weeks of CR	Δ of Exercise Capacity	*p*
Age (years)	<55	9	3.74 (3.0–6.5)	4.8 (3.5–6.1)	1.03 (−0.03–1.7)	0.0000001	49	5.26 (3.32–8.34)	6.4 (4.02–10.8)	1.17 (−0.14–3.0)	0.0000001
	≥55	97	4.02 (2.19–6.6)	4.5 (2.33–6.9)	0.03 (−1.2–3.14)	0.0000001	131	4.56 (2.68–7.08)	5.47 (3.21–7.76)	1.12 (−1.29–3.1)	0.0000001
BMI (kg/m^2^)	<25	23	4.4 (2.75–6.6)	4.8 (3.91–6.93)	0.01 (−0.04–1.9)	0.007	27	5.4 (2.6–8.3)	6.8 (4.3–10.8)	1.4 (−0.12–2.9)	0.00001
	≥25	83	3.87 (2.1–6.2)	4.5 (2.33–6.9	0.04 (−1.2–3.14	0.0000001	153	4.67 (3.01–7.1)	5.5 (3.2–7.9)	1.09 (−1.29–3.1)	0.0000001
LVEF (%)	≤40%	7	4.31 (3.58–5.28)	5.2 (3.55–6.9)	0.68 (−0.03–3.14)	0.08	18	4.35 (3.32–6.27)	5.2 (3.77–6.55)	1.1 (−0.02–1.46)	0.0008
	41%-49%	31	3.8 (2.3–6.1)	4.7 (2.3–6.9)	1.23 (−0.1–2.6)	0.0002	60	4.83 (2.68–6.14)	5.8 (3.21–8.24)	1.17 (−0.15–2.98)	0.0000001
	≥50%	68	4.03 (2.2–6)	4.4 (2.7–6.7)	0.02 (−1.2–1.5)	0.00003	102	4.74 (3.13–8.34)	5.6 (4.11–10.79)	1.12 (−1.29–3.1)	0.0000001
CAD	single-vessel disease	52	3.9 (2.3–6.5)	4.8 (2.3–6.9)	0.97 (−0.1–3.14)	0.0000001	64	4.85 (3.01–6.96)	5.8 (3.91–8.24)	1.1 (−0.15–3.1)	0.0000001
	two-vessel disease	31	4.0 (3.0–6.2)	4.2 (3.2–6.6)	0.01 (−1.2–2.26)	0.02	52	4.76 (3.26–6.2)	5.66 (3.21–7.5)	1.15 (−1.29–2.98)	0.0000001
	three-vessel disease	20	4.07 (2.1–6.6)	4.3 (2.7–6.9)	0.1 (−0.1–1.9)	0.19	59	4.63 (2.68–8.34)	5.5 (3.5–10.79)	1.14 (−0.14–2.7)	0.0000001

CAD—coronary artery disease; CR—cardiac rehabilitation; BMI—body mass index; LVEF—left ventricular ejection fraction.

## Data Availability

Not applicable.

## References

[1] WHO the Top 10 Causes of Death. https://www.who.int/en/news-room/fact-sheets/detail/the-top-10-causes-of-death.

[2] Anderson L., Oldridge N., Thompson D.R., Zwisler A.-D., Rees K., Martin N., Taylor R.S. (2016). Exercise-Based Cardiac Rehabilitation for Coronary Heart Disease. J. Am. Coll. Cardiol..

[3] Ambrosetti M., Abreu A., Corrà U., Davos C.H., Hansen D., Frederix I., Iliou M.C., Pedretti R.F., Schmid J.-P., Vigorito C. (2021). Secondary prevention through comprehensive cardiovascular rehabilitation: From knowledge to implementation. 2020 update. A position paper from the Secondary Prevention and Rehabilitation Section of the European Association of Preventive Cardiology. Eur. J. Prev. Cardiol..

[4] McDonagh T.A., Metra M., Adamo M., Gardner R.S., Baumbach A., Böhm M., Burri H., Butler J., Čelutkienė J., Chioncel O. (2021). Corrigendum to: 2021 ESC Guidelines for the diagnosis and treatment of acute and chronic heart failure: Developed by the Task Force for the diagnosis and treatment of acute and chronic heart failure of the European Society of Cardiology (ESC) With the special contribution of the Heart Failure Association (HFA) of the ESC. Eur. Heart J..

[5] Myers J., Prakash M., Froelicher V., Do D., Partington S., Atwood J.E. (2002). Exercise Capacity and Mortality among Men Referred for Exercise Testing. N. Engl. J. Med..

[6] Taylor R.S., Brown A., Ebrahim S., Jolliffe J., Noorani H., Rees K., Skidmore B., Stone J.A., Thompson D.R., Oldridge N. (2004). Exercise-based rehabilitation for patients with coronary heart disease: Systematic review and meta-analysis of randomized controlled trials. Am. J. Med..

[7] Rauch B., Davos C.H., Doherty P., Saure D., Metzendorf M.I., Salzwedel A., Völler H., Jensen K., Schmid J. (2016). The prognostic effect of cardiac rehabilitation in the era of acute revascularization and statin therapy: Systematic review and meta-analysis of randomized and non randomized studies—The Cardiac Rehabilitation Outcome Study (CROS). Eur. J. Prev. Cardiol..

[8] Jneid H., Fonarow G.C., Cannon C.P., Hernandez A.F., Palacios I.F., Maree A.O., Wells Q., Bozkurt B., Labresh K.A., Liang L. (2008). Sex differences in medical care and early death after acute myocardial infarction. Circulation.

[9] Feola M., Garnero S., Daniele B., Mento C., Dell’Aira F., Chizzolini G., Testa M. (2015). Gender differences in the efficacy of cardiovascular rehabilitation in patients after cardiac surgery procedures. J. Geriatr. Cardiol..

[10] Perera S., Aslam A., Stehli J., Kaye D., Layland J., Nicholls S.J., Cameron J., Zaman S. (2021). Gender Differences in Healthy Lifestyle Adherence Following Percutaneous Coronary Intervention for Coronary Artery Disease. Heart Lung Circ..

[11] Hao Y., Liu J., Liu J., Yang N., Smith Jr S.C., Huo Y., Fonarow G.C., Ge J., Taubert K.A., Morgan L. (2019). Sex Differences in In-Hospital Management and Outcomes of Patients With Acute Coronary Syndrome. Comp. Study Circ..

[12] Shaw L.J., Bugiardini R., Merz C.N.B. (2009). Women and Ischemic Heart Disease: Evolving Knowledge. J. Am. Coll. Cardiol..

[13] Reynolds H.R., Srichai M.B., Iqbal S.N., Slater J.N., Mancini G.B., Feit F., Pena-Sing I., Axel L., Attubato M.J., Yatskar L. (2011). Mechanisms of Myocardial Infarction in Women Without Angiographically Obstructive Coronary Artery Disease. Circulation.

[14] Vanhees L., Rauch B., Piepoli M., van Buuren F., Takken T., Börjesson M., Bjarnason-Wehrens B., Doherty P., Dugmore D., Halle M. (2012). Importance of characteristics and modalities of physical activity and exercise in the management of cardiovascular health in individuals with cardiovascular disease (Part III). Eur. J. Prev. Cardiol..

[15] Borg G.A. (1982). Psychophysical bases of perceived exertion. Med. Sci. Sports Exerc..

[16] Gottdiener J.S., Bednarz J., Devereux R., Gardin J., Klein A., Manning W.J., Morehead A., Kitzman D., Oh J., Quinones M. (2004). American Society of Echocardiography recommendations for use of echocardiography in clinical trials. J. Am. Soc. Echocardiogr..

[17] Kodama S., Saito K., Tanaka S., Maki M., Yachi Y., Asumi M., Sugawara A., Totsuka K., Shimano H., Ohashi Y. (2009). Cardiorespiratory fitness as a quantitative predictor of all-cause mortality and cardiovascular events in healthy men and women: A meta-analysis. JAMA.

[18] Myers J. (2014). New American heart association/American college of cardiology guidelines on cardiovascular risk: When will fitness get the recognition it deserves?. Mayo Clin. Proc..

[19] Imboden M.T., Harber M.P., Whaley M.H., Finch W.H., Bishop D.L., Kaminsky L.A. (2018). Cardiorespiratory Fitness and Mortality in Healthy Men and Women. J. Am. Coll. Cardiol..

[20] Beckie T.M., Beckstead J.W., Kip K., Fletcher G. (2013). Physiological and Exercise Capacity Improvements in Women Completing Cardiac Rehabilitation. J. Cardiopulm. Rehabil. Prev..

[21] Sattelmair J., Pertman J., Ding E.L., Kohlll H.W., Haskell W., Lee I.M. (2011). Dose response between physical activity and risk of coronary heart disease: A meta-analysis. Circulation.

[22] Araya-Ramírez F., Briggs K.K., Bishop S.R., Miller C.E., Moncada-Jiménez J., Grandjean P.W. (2010). Who Is Likely to Benefit From Phase II Cardiac Rehabilitation?. J. Cardiopulm. Rehabil. Prev..

[23] Ghashghaei F.E., Sadeghi M., Marandi S.M., Ghashghaei S.E. (2012). Exercise-based cardiac rehabilitation improves hemodynamic responses after coronary artery bypass graft surgery. ARYA Atheroscler..

[24] Kavanagh T., Hamm L.F., Beyene J., Mertens D.J., Kennedy J., Campbell R., Fallah S., Shephard R.J. (2008). Usefulness of Improvement in Walking Distance Versus Peak Oxygen Uptake in Predicting Prognosis After Myocardial Infarction and/or Coronary Artery Bypass Grafting in Men. Am. J. Cardiol..

[25] Gupta R., Sanderson B.K., Bittner V. (2007). Outcomes at one-year follow-up of women and men with coronary artery disease discharged from cardiac rehabilitation: What benefits are maintained?. J. Cardiopulm. Rehabil. Prev..

[26] Gee M.A., Viera A.J., Miller P.F., Tolleson-Rinehart S. (2014). Functional Capacity in Men and Women Following Cardiac Rehabilitation. J. Cardiopulm. Rehabil. Prev..

[27] O’Farrell P., Murray J., Huston P., Legrand C., Adamo K. (2000). Sex differences in cardiac rehabilitation. Can. J. Cardiol..

[28] Araya-Ramírez F., Moncada-Jiménez J., Grandjean P.W., Franklin B.A. (2021). Improved Walk Test Performance and Blood Pressure Responses in Men and Women Completing Cardiac Rehabilitation: Implications Regarding Exercise Trainability. Am. J. Lifestyle Med..

[29] Colbert J.D., Martin B.-J., Haykowsky M.J., Hauer T.L., Austford L.D., Arena R.A., Knudtson M.L., Meldrum D.A., Aggarwal S.G., Stone J.A. (2015). Cardiac rehabilitation referral, attendance and mortality in women. Eur. J. Prev. Cardiol..

[30] Grodstein F., Stampfer M. (1995). The epidemiology of coronary heart disease and estrogen replacement in postmenopausal women. Prog. Cardiovasc. Dis..

[31] Grodstein F., Manson J.E., Colditz G.A., Willett W.C., Speizer F.E., Stampfer M.J. (2000). A Prospective, Observational Study of Postmenopausal Hormone Therapy and Primary Prevention of Cardiovascular Disease. Ann. Intern. Med..

[32] Hulley S., Grady D., Bush T., Furberg C., Herrington D., Riggs B., Vittinghoff E. (1998). Randomized Trial of Estrogen Plus Progestin for Secondary Prevention of Coronary Heart Disease in Postmenopausal Women. JAMA.

[33] Rossouw J.E., Anderson G.L., Prentice R.L., LaCroix A.Z., Kooperberg C., Stefanick M.L., Jackson R.D., Beresford S.A., Howard B.V., Johnson K.C. (2002). Risks and benefits of estrogen plus progestin in healthy postmenopausal women: Principal results from the women’s health initiative randomized controlled trial. JAMA.

[34] De Smedt D., De Bacquer D., De Sutter J., Dallongeville J., Gevaert S., De Backer G., Bruthans J., Kotseva K., Reiner Ž., Tokgözoğlu L. (2016). The gender gap in risk factor control: Effects of age and education on the control of cardiovascular risk factors in male and female coronary patients. The EUROASPIRE IV study by the European Society of Cardiology. Int. J. Cardiol..

[35] Mosca L., Linfante A.H., Benjamin E.J., Berra K., Hayes S.N., Walsh B.W., Fabunmi R.P., Kwan J., Mills T., Simpson S.L. (2005). National Study of Physician Awareness and Adherence to Cardiovascular Disease Prevention Guidelines. Circ..

[36] Gu Q., Burt V.L., Paulose-Ram R., Dillon C.F. (2008). Gender Differences in Hypertension Treatment, Drug Utilization Patterns, and Blood Pressure Control Among US Adults With Hypertension: Data From the National Health and Nutrition Examination Survey 1999–2004. Am. J. Hypertens..

[37] Bird C.E., Fremont A.M., Bierman A.S., Wickstrom S., Shah M., Rector T., Horstman T., Escarce J.J. (2007). Does quality of care for cardiovascular disease and diabetes differ by gender for enrollees in managed care plans?. Womens Health Issues.

[38] Lim S.K., Han J.Y., Choe Y.R. (2016). Comparison of the Efects of Cardiac Rehabilitation between Obese and Non-obese Patients After Acute Myocardial Infarction. Ann. Rehabil. Med..

[39] Piepoli M.F., Davos C., Francis D.P., Coats A.J. (2004). ExTraMATCH Collaborative Exercise training meta-analysis of trials in patients with chronic heart failure (ExTraMATCH). BMJ.

[40] O’Connor C.M., Whellan D.J., Lee K.L., Keteyian S.J., Cooper L.S., Ellis S.J., Leifer E.S., Kraus W.E., Kitzman D.W., Blumenthal J.A. (2009). Efficacy and safety of exercise training in patients with chronic heart failure: HF-ACTION randomized controlled trial. JAMA.

[41] Aguiar R.S., Abreu A., Soares R.M., Rio P., Filipeb C., Rodriguesa I., Monteiro A., Soaresa C., Ferreira V., Silvaa S. (2017). Cardiac rehabilitation after acute coronary syndrome: Do all patients derive the same benefit?. Rev. Port. Cardiol..

[42] Flynn K.E., Pina I.L., Whellan D.J., Lin L., Blumenthal J.A., Ellis S.J., Fine L.J., Howlett J.G., Keteyian S.J., Kitzman D.W. (2009). Effects of exercise training on health status in patients with chronic heart failure: HF-ACTION randomized controlled trial. JAMA.

[43] Taylor R.S., Walker S., Ciani O., Warren F., Smart N.A., Piepoli M., Davos C.H. (2019). Exercise-based cardiac rehabilitation for chronic heart failure: The EXTRAMATCH II individual participant data meta-analysis. Health Technol. Assess..

[44] Trachsel L.-D., Boidin M., Henri C., Fortier A., Lalongé J., Juneau M., Nigam A., Gayda M. (2021). Women and men with coronary heart disease respond similarly to different aerobic exercise training modalities: A pooled analysis of prospective randomized trials. Appl. Physiol. Nutr. Metab..

[45] Gerlach S., Mermier C., Kravitz L., Dalleck L., Zuhl M. (2021). Predicting functional outcomes among CAD who complete cardiac rehabilitation. Int. J. Cardiol. Cardiovasc Dis..

